# Prevalence of visual dysfunction and ocular motility disorders in developmentally delayed patients

**DOI:** 10.12669/pjms.39.6.7328

**Published:** 2023

**Authors:** Muhammad Ajmal Ch, Muhammad Ajmal Chaudhary, Muhammad Nishat Bukhari, Naima Ahmed

**Affiliations:** 1 Muhammad Ajmal Ch, M Phil Orthoptics Department of Pediatric Ophthalmology, The University of Child Health, Lahore, Pakistan; 2 Muhammad Ajmal Chaudhary, FCPS, FPO Department of Pediatric Ophthalmology, Sheikh Zayed Medical College, Rahim Yar Khan, Pakistan; 3 Muhammad Nishat Bukhari, M Phil Optometry Scholar Department of Ophthalmology Shalamar Institute of Health Sciences, Lahore, Pakistan; 4 Naima Ahmed, BSc Hons Optometry and Orthoptics Department of Ophthalmology Shalamar Institute of Health Sciences, Lahore, Pakistan

**Keywords:** Developmentally delayed, Refractive errors, Ocular motility, Visual dysfunction, Contrast sensitivity

## Abstract

**Objective:**

To evaluate the prevalence of reduced visual acuity (VA), refractive errors (RE), reduced contrast sensitivity and strabismus in developmentally delayed (DD) patients.

**Methods:**

This descriptive cross sectional study was carried out in Ophthalmology Departments of Mayo Hospital, Lahore, The Children’s Hospital, Lahore and The Children’s Hospital, Multan from June 2019 to December 2019. We recruited 257 patients of either gender, between the ages of 06-16 years having intelligence quotient (IQ) ≤ 80 by Wechsler Intelligence Scale for Children (WISC) from the out-patient departments. Detailed systemic and ophthalmic history was taken and through anterior and posterior segment examination was carried out. VA was assessed with age matched VA charts. Cycloplegic refraction with 1% cyclopentolate was carried out. Contrast sensitivity was measured with hiding Heidi charts. Strabismus was assessed with Hirschberg and covers /uncover tests.

**Results:**

The mean age of the patients was 8.88 years with standard deviation (SD) of ± 2.70. The prevalence of reduced VA, RE, strabismus and reduced contrast sensitivity in these children were 43.58%, 52.92%, 52.14% and 32.7% respectively. Out of these 52.92% RE, 56 (21.79%) were myopic, 66 (25.68%) were hyperopic and 14 (05.45%) were astigmatic. The percentage of esotropia was 72 (28.02%) and exotropia was 62 (24.12%).

**Conclusion:**

The results of our study in DD children have shown that a significant number of children have reduced VA, RE, strabismus and reduced contrast sensitivity. Apart from general management of DD children by a pediatrician, the ophthalmic management of these problems must be carried out by a pediatric ophthalmologist to improve their quality of life.

## INTRODUCTION

A child learns variable skills during his growth and development such as speaking, happy for the first time or saying goodbye by waving hands. These are known as fundamental growth milestones. A developmentally delayed (DD) child does not achieve developmental milestones in comparison to his age matched peers.^1^ Delayed development in childhood is associated with poor educational attainment and low income during adult life, leading to poverty.^2^ It is an important public health problem in low and middle income countries, like Pakistan. Vision and visual perception play a vital role in normal child development and is crucial to mental developmental.^3^

Vision is essential to acquire skills such as language, facial interpretation, and skills requiring coordination of the eye with the hand. Any specific problem of vision related to learning in DD children not only affects the process of visual input but also affects visual processing and integration. RE are major cause of treatable and avoidable blindness in the world. Its prevalence varies with age, gender, geographical distribution, and ethnicity and also depends on ongoing progress in eye care services.^4^ High myopia is more likely associated with threatening visual impacts than moderate or low myopia (high myopia > -6.0D, moderate myopia -3.0D to -6.0D and low myopia -0.75D to -3.0D).^5^

The failure of normal eye coordination may leads to the development of strabismus and amblyopia in children.^6^ There is a strong association between uncorrected hyperopia, abnormal visual development and learning disability.^7^ Strabismus in children is a common eye disorder with a frequency range from 0.8%-5.65%, in Western countries overall prevalence is 02%-06%^8^ and in a study conducted in Pakistan the frequency of strabismus was 6.21%.^9^ Untreated strabismus can lead to lowered binocular single vision and amblyopia and finally lead to psychosocial problems such as decreased self-confidence, mood disorder, lower interpersonal relationships and decreased employment.^10^,^11^

The most common ocular disorders in DD children are cerebral visual impairment, RE, strabismus, amblyopia, keratoconus, cataract, optic atrophy, optic coloboma, Duane retraction syndrome, blepharoptosis and nasolacrimal duct stenosis.^12^ The main problem for these DD children appears to be the inability to express their eye problems appropriately to their parents. Therefore such children should receive a complete eye examination. A significant number of DD children visit the pediatric eye hospital. It is important to know the frequency of squint, RE and decreased contrast sensitivity to help these children optimally.

Every child is special but “Special child” needs special attention. Everyone has a right to have a healthy life and to protect this right, it is important to take early intervention and treatment. That is why the systemic and comprehensive ophthalmic examination is necessary for these patients. This study was designed to evaluate prevalence of visual dysfunction and ocular motility disorders in DD children. Currently, in Pakistan, little literature is available regarding ocular problems in DD children. Our study will highlight this important issue, will enhance the knowledge of eye care professionals like ophthalmologists, optometrists and orthoptists and will contribute to add information to the existing knowledge along with human and economic resources planning, policy making and further research.

## METHODS

This descriptive cross sectional study was carried out in Ophthalmology Departments of Mayo Hospital, Lahore, The Children’s Hospital, Lahore and The Children’s Hospital, Multan from June 2019 to December 2019. A total of 257 patients were included by non-probability convenient sampling by using 95% confidence level, 6% absolute precision with expected perecnetage of contrast sensitivity as 40%.^11^

### Ethical Approval:

The proposal was approved from the ethical review board (No. 720/RC/KEMU, Date: 04-05-2019).

Patients of both gender, between the ages of 6-16 years and having intelligence quotient (IQ) ≤ 80 by Wechsler Intelligence Scale for Children (WISC) were included. While patients not fulfilling the inclusion criteria and patients with other ocular pathologies like congenital glaucoma, congenital cataract, macular or retinal dystrophies etc were excluded. The whole procedure was explained to the participant / parents before taking the informed consent. A detailed systemic and ophthalmic history regarding age, duration of symptoms and presenting complaints was taken.

A through anterior and posterior segment examination was carried out. Log MAR VA chart, Kay pictures, Snellen VA chart and E charts were used for vision assessment. Cycloplegic refraction was performed using 1% cyclopentolate. Contrast sensitivity was measured with hiding Heidi Charts. Strabismus was examined with Hirschberg and covers / uncover tests. Data were entered with Statistical Package for Social Science (SPSS) version 20.0. The quantitative variable like age was presented in the form of mean ± SD. Qualitative variables like gender, VA, RE, contrast sensitivity and strabismus were presented as frequencies and percentages.

## RESULTS

**Fig.1:**
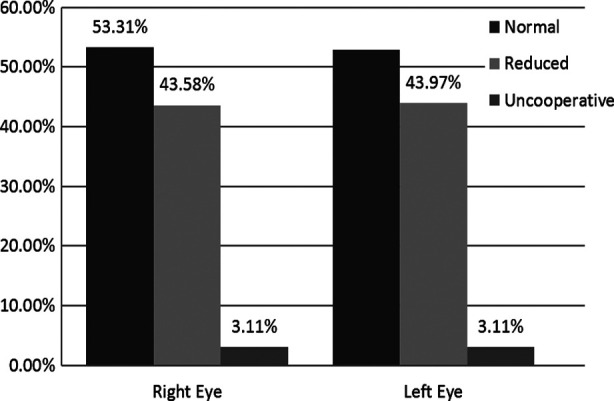
Descriptive statistics of right and left eye VA.

A total of 257 patients were included in the study with the age range of 06-16 years (mean age 8.88 ± 2.70 years), 160 (62.3%) were males and 97 (37.7%) were females. Out of 257 patients, eight patients (3.11%) were not able to participate in VA testing. So, VA testing of 249 (96.89%) patients was done. In the right eye normal VA was seen in 137 (53.31%) patients and 112 (43.58%) patients showed reduced VA whereas in the left eye normal VA was seen in 136 (52.92%) patients and 113 (43.97%) patients showed reduced VA as shown in the Fig-1.

There was no significant difference between the right and left eye VA. Regarding refractive status of our 257 patients, 136 (52.92%) patients showed RE and 121(47.08%) patients were emmetropic. Out of 136 patients with RE, 56 (21.79%) were myopic, 66 (25.68%) were hyperopic and 14 (05.45%) were astigmatic. Contrast sensitivity of our 257 patients revealed that 20 (7.8%) were not able to participate in this testing. Normal contrast sensitivity was seen in 153 (59.5%) patients while 84 (32.7%) showed reduced contrast sensitivity. Strabismus was seen in 134 patients (52.14%) whereas 123 (47.86%) patients were orthotropic. Out of 134 patients with strabismus, esotropia was seen in 72 (28.02%) patients and exotropia was seen in 62 (24.12%) patients. Other ocular motility disorders like vertical deviations and inferior oblique overaction were not observed.

## DISCUSSION

According to our results the prevalence of reduced VA, RE, strabismus and reduced contrast sensitivity in these children were 43.58%, 52.92%, 52.14%, and 32.7% respectively. Out of these 52.92% RE, 56 (21.79%) were myopic, 66 (25.68%) were hyperopic and 14 (05.45%) were astigmatic. The percentage of esotropia was 72 (28.02%) and exotropia was 62 (24.12%).

When a child has an intelligence quotient (IQ) level less than 80 and is not able to achieve required growth milestones as compared to his age matched children, is termed as DD or global developmental delay (GDD). Prevalence of childhood disabilities is up to 10% of the total pediatric population and this figure is much higher in Pakistan.^13^ Early childhood is a period of great opportunity for optimal brain growth and is a period of vulnerability as well. Developmental domains like language, motor, vision, speech, hearing, cognition and socio-emotional behaviour rapidly occurs during this period. These developmental domains do not develop in isolation, rather enable each other and interact mutually as the child learns to become more independent. For example, as a child learns to see, he will increasingly reach for and play with objects and will develop coordination and motor skills.^14^

We evaluated the distribution and percentage of reduced VA, RE, strabismus and reduced contrast sensitivity in 257 DD children having IQ ≤ 80 by WISC, between the age ranges of 06-16 years, mean age was 8.88 ± 2.70 years. In our study, male children were more 160 (62.3%) than female 97 (37.7%), similar to the studies conducted by Smitha KS et al.^15^ and Afroze R et al.^16^

Vora et al. conducted a study on DD children and reported the prevalence of RE as 58.5%. The prevalence rates were 18.6%, 24.3% and 27.1% for hyperopia, myopia, and astigmatism respectively. The prevalence of reduced contrast sensitivity was 50%.^17^ Vora study had similar results of RE and contrast sensitivity as our study had RE 52.92% and contrast sensitivity 32.7%. Reena et al. reported the prevalence of RE and strabismus 41.3% and 40% respectively in children with delayed milestones of six months to three years of age.^18^ Reena study had similar results of RE and strabismus as our study had RE 52.92% and strabismus 52.14% except the age being six months to three years. Nielsen et al. conducted a study on 1126 DD children whose age range was 4-15 years. Overall prevalence of strabismus was 26.8%, esotropia 14.9%, exotropia 10.3% and mixed type 1.6%. The prevalence of RE was hyperopia 15.3%, myopia 10.8% and astigmatism 20.6%.^19^ Nielsen study had similar results of RE as our study had RE 52.92% and the prevalence of strabismus is varied may be due to the difference in population size and other measuring methods.

Yekta AA et al. conducted a study on 406 children with intellectual disability and reported that 78.6% of patients were emmetrope, 14.5% were myopic, 6.9% were hypermetropic and 18.5% were astigmatic. While 89.9% of children had orthophoria, 1.0% had esophoria, 6.4% had exophoria and 2.2% had suppression. The best corrected VA achieved in the right eye was 98.5% and 98% in the left eye.^20^ Abbass study had almost similar results of RE as our study showed 52.92% but the prevalence of strabismus and VA is varied may be due to the difference in population size and other measuring methods.

Joshi et al. conducted a study on 112 DD children age range was 02-12 years and showed the frequency of all ocular disorders as 84.8%. RE were most predominant with a frequency of 79.5% and strabismus was 46.4%. Astigmatism was 44.6%, hyperopia was 21.9% and myopia was 12.1%. The frequency of exotropia was 52% and esotropia was 48%.^21^ Joshi study had similar results of strabismus as our study showed 52.14% but have higher prevalence of RE may be due to small sample size and other measuring methods.

Smitha KS et al. in another study examined 113 consecutive children with 01-15 years of age visiting the OPD of ophthalmology department. They concluded the prevalence of RE 75% with a higher incidence of hypermetropia in intellectually disabled children.^22^ Smitha study had different prevalence of RE than our study due to the difference in age range and sample size.

Gogate P et al. examined 664 students with learning disabilities, 526 patients was greater than 16 years of age. They reported that children having learning disabilities had 45.3% ocular disorders. Untreated RE were seen in 27.3% patients, strabismus in 15.8%, nystagmus in 6.8%, optic atrophy was seen in 6.5% patients and 2.5% patients had inborn anomalies.^23^ Gogate study had different prevalence of RE and strabismus from our study due to the difference in age range, sample size and race.

Qayyum A et al. conducted a recent study in Pakistan in DD children. They included 406 children with male preponderance of 63% and 41% were in the age range of 1-2 years. RE were the most common ocular problem (30.29%), of which hyperopia was seen in 70.7% and myopia in 29.3% patients. Strabismus was seen in 15.5% of children, esotropia (65%) being more prevalent than exotropia (34.9%).^24^ This study had different prevalence of RE and strabismus than our study, this difference could be due to elder age group and small sample size in our study.

Our study revealed that a significant number of patients had reduced VA, RE, strabismus and reduced contrast sensitivity which may affect child growth and education. Most of these disorders in DD children can be treated successfully if timely diagnosed, giving these children a chance to develop better physically, mentally, socially and academically. Currently, in Pakistan, little literature is available regarding ocular problems in DD children. Our study will add to the already published international research evidence on this topic.

### Limitations of the study:

It is mainly a pediatric age group study, so our results are not a ture picture of general population. We included relatively older children and our sample size was relatively small. Further research is needed at provincial and national levels to address this highly important issue.

## CONCLUSIONS

Our study revealed that a significant number of DD patients had reduced VA, RE, strabismus and reduced contrast sensitivity. Apart from general management of DD children, their ophthalmic management of these problems must be carried out to improve their quality of life. Strengthening relations and teamwork is needed between child development, community pediatric service, pediatric ophthalmic care and child specialist education services to meet the visual needs of these children. Annual DD patient’s eye screening must be taken at the national level in all rehabilitation centers, special schools and hospitals. This ensures early detection and timely treatment of visual and ocular eye problems in these special children.

### Authors Contribution:

**MAC:** Did data collection, Statistical analysis and prepared the manuscript.

**MAC:** Conceived, designed and did editing of manuscript, is responsible for integrity of research.

**MNB:** Did data collection, interpretation of data and manuscript writing.

**NA:** Did review and final approval of manuscript.
